# Associative Obligation and the Social Contract

**DOI:** 10.1007/s11406-016-9797-5

**Published:** 2017-01-05

**Authors:** Albert Weale

**Affiliations:** Department of Political Science, School of Public Policy, University College London, 29/30 Tavistock Square, London, WC1H 9QU UK

**Keywords:** Political obligation, Social contract theory, Political theory methods

## Abstract

John Horton has argued for an associative theory of political obligation in which such obligation is seen as a concomitant of membership of a particular polity, where a polity provides the generic goods of order and security. Accompanying these substantive claims is a methodological thesis about the centrality of the phenomenology of ordinary moral consciousness to our understanding of the problem of political obligation. The phenomenological strategy seems modest but in some way it is far-reaching promising to dissolve some long-standing problems of political theory. However, it fails at just the point at which a theory of political obligation is needed, namely when individuals question the grounds of their political obligation. A principle of obligation is needed to provide individuals with a reason for compliance with authoritative social rules when the exercise of that obligation is irksome. It is at this point that we need to invoke the idea of society as an implicit social contract, in which obligations are seen as stemming from those terms that it would be in the interests of individuals to agree in a social contract. This is consistent with the method of reflective equilibrium.

In *Political Obligation* and related papers John Horton has set out an associative account of political obligation. The account is made up of two principal claims. The first is “that political obligations are a concomitant of membership of a particular polity”. The second is that a polity is “a form of association that has as its generic value the goods of order and security” (Horton 2010: 191; see also, Horton 2006, 2007). According to Horton, the conjunction of these two propositions gives the core of a satisfactory theory of political obligation. Each proposition is justified by informal empirical arguments that run as follows. As a matter of fact, most people, at least those living in stable polities, think of themselves as having a special connection with the society in which they were born and grew up. This social fact makes political obligation a concomitant of membership of a particular polity. Secondly, polities are forms of association with the unique ability to supply the goods of order and security. So, the associative theory rests upon two social facts that explain why at least some polities are the objects of obligation. People do feel a special connection with their particular polity and order and security are generally goods for human beings, obtainable only through political organisation. According to Horton, it is this union of the particular identification and the general benefit that is required in a theory of political obligation. Obligation is neither voluntary nor owed to the world at large. Instead it is incurred as an incident of membership and is owed to a particular polity (Horton 2010: 148).

Any proponent of such a theory has to confront a number of tricky issues involved in its articulation. For example, there is an important distinction between institutional and moral obligations, and how political obligation is related to the latter has to be spelt out. Also, any such theory has to specify how far beyond the scope of simple legal obedience runs the writ of political obligation, as well as why we have in associative theory an account of actual, rather than merely subjectively perceived, obligations. However, I do not propose to discuss these issues in any detail in this paper. This is largely because Horton’s own exposition of the theory deals with them plausibly, rebutting some obvious objections, thereby strengthening his own account. So, rather than be concerned with the substance of Horton’s associative theory, I shall be principally concerned with issues of method. Horton’s articulation of an associative theory of obligation is accompanied by some interesting and thoughtful methodological claims seeking to underpin his own distinctive substantive thesis. And it is to these methodological claims that my attention will be principally directed.

However, although the focus will be upon method, the analysis is intended to lead to a substantive conclusion, namely that a satisfactory theory of obligation will have to supplement the two core propositions of Horton’s theory with an account, drawn from social contract theory, of the reasons that can be offered to individuals explicating why they should play their part in any scheme of cooperation necessary to produce the social goods of law and order from which they benefit. Political association has the distinctive value for individuals by virtue of its ability to supply of law and order, but individuals can often secure those benefits without incurring the costs that the fulfilment of political obligations brings in its wake. In this situation reasons need to be given to individuals explaining why they need to participate in providing those goods. When the need for such reasons arises, the phenomenological method that is at the centre of Horton’s argumentative strategy invokes a theory beyond itself.

Why focus so much on method? One answer is that Horton himself is methodologically self-aware, offering his approach as an interpretative theory. Although he does not present an extended abstract argument in favour of his approach, we are invited to see his account of political obligation as growing out of a reflective interpretation of ordinary moral consciousness combined with a few elementary truths about the goods that only a political community can provide. His methodological programme is largely worked out through exhibition rather than sustained exposition in an abstract form. However, there are, throughout the text of *Political Obligation*, a number of important remarks on method. In Horton’s account of political obligation, then, we are invited to see the value of the method through its utility in solving a particularly well-known and intractable problem.

Horton’s interpretative method can be placed in a broader characterisation of styles of reasoning in political theory. Those styles of reasoning may be placed on a scale running from the demonstrative to the oracular. At the demonstrative end are those theorists who work from a limited number of assumptions – assumptions that may be taken as self-evident, axiomatic or the core of a particular value system – from which they derive conclusions through a deductive process of reasoning (for just one example, see Gauthier 1986). Formal social choice theorists also fall into this category, as do those normative theorists who aspire to fact-insensitivity in their theorizing. At the opposite end of the spectrum are oracular theorists, of whom Nietzsche is the paradigm. Oracular theorists are in the business of expressing discontent with dominant intellectual paradigms, and they are therefore hostile to forms of argument that concede to readers the capacity to reason demonstratively to a desired conclusion. Instead, their self-appointed task is to produce a vivid picture of an alternative way of conceiving political obligations and relations. At the opposite end of the scale from the deductive mode of theorising, their style is aphoristic, poetical, provocative. When Nietzsche (1889: 23) wrote in *Twilight of the Idols* that man does not strive for happiness, only the Englishman does, it would be missing the point to treat this purely as an empirical claim of the sort that could be checked by examining the World Values Survey. He was seeking to provoke a reaction, shocking people into a transvaluation of values consequent upon the demise of religion.

Of one methodological truth we can be sure. There is a large range of options between the extremes represented by these ends of the spectrum. Horton’s approach is one of the most interesting of these intermediate options. It shares with the oracular approach a scepticism about the reach and scope of deductive reasoning, seeing at crucial places the need to put a certain picture before our minds rather than set out a chain of inference. Its motto might be Wittgenstein’s (1968: § 115) famous remark in the *Philosophical Investigations*: “A picture held us captive. And we could not get outside it, for it lay in our language and language seemed to repeat it to us inexorably.” Some technique other than that of deductive demonstration is needed to rid us of the wrong picture. However, by contrast with the oracular approach, Horton’s method shows a preference for seduction over provocation. It invites readers to reflect upon a certain set of assumptions that they take for granted, and then offers an explication of those assumptions in a form intended to entice those readers towards a particular conclusion.

In this enterprise Horton relies upon the method of reflection, but not the method of reflective equilibrium. Any theory that takes ordinary moral consciousness as its starting-point will have to use the method of reflection, drawing out the elements of common sense morality that are thought important. However, such reflection need not involve reflective equilibrium. The latter is a method central to the Sidgwick/Rawls programme of offering a general theory, be it utilitarian or contractualist, that seeks to account for the principles of common sense morality in terms of an intellectual construction that shows how those principles can be seen as deductions from that construction (Rawls 1996: 89–90; Rawls 1999: 18–19; Sidgwick 1907: Book IV). Unabashedly demonstrative and provocatively oracular theories both dispense, for their own reasons, with any attempt to anchor normative arguments in an understanding of ordinary moral consciousness, whether in reflection or reflective equilibrium. (As well as the example of Nietzsche, compare Gauthier 1986: 269, who explicitly rejects a place for reflective equilibrium in his theorising). Horton denies that ordinary moral consciousness can be rejected, but he also denies that the method of reflective equilibrium is necessary. The content of ordinary moral consciousness provides a constraint upon what we can say in our theories. Yet, although the theory is anchored in reflection, it does not aim at reflective equilibrium. Reflective equilibrium presupposes that we can formulate a relationship between an abstract general theory, on the one hand, and the contents of ordinary moral consciousness on the other, and Horton rejects the appeal of abstract theory. His is a particular conception of what a normative theory is, and on that conception there is no abstract theory to come into relation with moral consciousness. Theory merely explicates ordinary moral consciousness.

## The Methodological Stance

Horton states his preferred methodological position towards the beginning of *Political Obligation* in a passage I shall quote at length:

Much of contemporary political philosophy is strongly normative. That is, it sees itself as primarily in the business of seeking moral justification for political institutions, practices or principles. It also tends to operate with a particular model of what a moral justification should look like. Typically, it is thought that moral justifications need to be grounded in first principles that are intuitively appealing or that are part of a broader picture that convincingly hangs together…. In what follows I do not entirely depart from a weaker reading of what this involves, but I also see the theoretical or philosophical task as more one of interpretation, more ‘hermeneutical’, than a matter of justification from first principles. Interpretation can itself be understood as a kind of justification, and to some extent that tends to be how it plays out in the subsequent argument. However, it is a rather more informal and relaxed conception of justification than many philosophers would find acceptable. My preferred designation is that what is sought is a philosophical or theoretical explanation. For my guiding thought is that if we can make sense of the idea of political obligation in a way that is intellectually coherent and morally unobjectionable then this constitutes a convincing theoretical or philosophical explanation of it. (Horton 2010:9).

Horton takes one central implication of this approach to be that any account of political obligation “should seek to accommodate the central features of ordinary thought about these matters”, “especially if its central features are widespread across may different societies and deeply embedded in social life” (Horton 2010: 9). He also urges that his theory meets the other requirements that any theory of political obligation is ordinarily thought to require. In particular: it should explain the standard case, where people are born into a polity; it should deal with cases where obligation is significant; and it should meet the requirement of showing how political obligation is owed to a particular polity (Horton 2010: 10).

Horton’s methodological thesis raises an interesting second-order question about how we are to determine the plausibility of the method itself. What is the right method for evaluating the force and reach of this method? Are we, for example, supposed to be as relaxed about this evaluation as Horton says we should be about our understanding of first-order justification? Could we, for example, simply say that another method would produce a different, but equally plausible, theory of political obligation? To this last question, the answer has to be “no”. If the methodological thesis is right, then no theory that does not square with ordinary moral consciousness can be justifiable. Yet, such a methodological constraint has implications for how we are to think about the substantive content of a political theories, and not just in relation to political obligation. To see why, consider the case of economic justice. Suppose we accept that, in the ordinary moral consciousness about economic justice as revealed by opinion surveys, considerations of desert figure strongly (see Miller 1999: chapter 4). Should we require our theories of economic justice to give a central place to the idea of desert? Horton’s methodological approach implies a positive answer to this question. Yet, a wide range of contemporary theories of economic justice are sceptical about the notion of desert (Barry 1995; Gauthier 1986; Hayek 1976: 72; Rawls 1999: 274; even Sadurski 1985, might be regarded as ultimately a view based on the principle of proportionality rather than desert). So, if we adopt the interpretative method, on the model recommended by Horton, and say that we must anchor our normative theories in ordinary moral consciousness, we should reject all the major current theories of economic justice on methodological grounds alone. In this respect, method constrains content. Conversely, if we regard Horton’s discussion of political obligation as some sort of demonstration project for the interpretative method, then anyone who finds the content of associative theory attractive will have difficulty detaching it from the method used to justify the theory. If content does not exactly vindicate method, it comes close to doing so.

Determining the right method for evaluating a method threatens an infinite regress. Avoiding this problem is like swimming in a cold sea: it is sometimes better to plunge in briskly rather than agonise on the shore about the water’s temperature. So here is the outline of my brisk plunge. Firstly, after clarifying the notion of “explanation” in Horton’s argument, I shall say something about the role the phenomenological method plays in his associative theory, drawing attention to what may be thought of as an anomalous relationship between the presuppositions of that method and the occasions on which the problem of political obligation will typically arise. That leads, secondly, to an exploration of what it means to provide reasons to agents for their political obligations in situations in which the phenomenology of obligation cannot be taken for granted. Finally, I shall offer a sketch of one version of a social contract approach that is intended to provide an essential element in our understanding of political obligation.

## The Phenomenological Strategy

To understand the phenomenological strategy it is helpful to understand what an interpretative explanation is. For that notion, let us return to three crucial sentences in Horton’s methodological manifesto:

Interpretation can itself be understood as a kind of justification, and to some extent that tends to be how it plays out in the subsequent argument. However, it is a rather more informal and relaxed conception of justification than many philosophers would find acceptable. My preferred designation is that what is sought is a philosophical or theoretical explanation. (Horton 2010: 9)

So the crucial equation being made here is that between “interpretation” and “a philosophical or theoretical explanation”. How are we to understand this relation?

In philosophical approaches to explanation influenced by empiricism the term “explanation” is contrasted with interpretation. The principal reason for making this distinction is that, on standard empiricist views of explanation, explanatory variables relate only contingently to explained variables. *Explanans* and *explanandum* are logically distinct. If they were not, we should merely have redescription rather than explanation. By contrast, in an interpretation, an interpreted item stands in a relationship of logical equivalence to its original. If I say that morphine’s “dormative powers” implies that morphine sends you to sleep, I have simply used two different ways of saying the same thing, rather than providing an explanation of why and how morphine works as it does. By contrast, if I say “an increase in demand induces an increase in price unless there is a corresponding increase in supply”, I have made a statement with genuine empirical content: there is a possible world in which the statement could be false. On the empiricist account of explanation, there should be no logical connection between *explanans* and *explanandum*. Rather to explain an event we need to show how the event is the product of a law-like non-logical relationship between two variables under given initial conditions. (For the classic exposition of this nomological account of explanation, see Nagel 1961: Chapter 4.)

To those influenced by the work of Wittgenstein, this non-logical relationship between *explanans* and *explanandum* has always seemed inappropriate in the case of human action. If, when I am thirsty, I believe that a glass of water will quench my thirst, then my drinking of the water seems more like a logical implication of my state of belief than it is the association of two independent states of affairs. It is partly for this reason that von Wright (1971) distinguished explanation from understanding. Understanding provides us with the reasons that make an agent act according to the logic of the practical inference, in which an action is like the conclusion of a syllogism. What Horton calls “philosophical or theoretical explanation” is, I suggest, von Wright’s “understanding”, meaning by this an explication of the meaning of concepts and their implications as those concepts shape the practical reasoning of agents. This is a form of logical analysis, although, in explicating the meaning of concepts for actors, empirical claims about what those actors believe will always be presupposed, as they are in Horton’s analysis. The concepts of obligation with which he is concerned are presumed to be present in the mental life of the members of a society.

This leads Horton to the adoption of the phenomenological method. The phenomenological method provides a way of meeting the particularity condition, which is one of the conditions that Horton says any adequate theory of political obligation must satisfy. The particularity condition requires that any theory of political obligation should show why it is that the members of a polity have an obligation to their own polity but not to any other. The force of the phenomenological method for Horton is that it reveals the fact that people do feel a special association with their polity. They are conscious of themselves as members of their polity. This does not mean that they are complacent or content with the actions or policies of their polity. Indeed, their membership may lead them to be especially critical of their polity’s government, particularly if that government acts in the name of the polity but contrary to the reflective convictions of (at least) some members of the polity. Nonetheless, it is central to the interpretative approach that it identifies those concepts that are to be found in the shared understanding of the bulk of the members of a particular society.

From one point of view, the phenomenological method embodies a modest intellectual strategy. Rather than focus upon a theoretical idea of seemingly doubtful cogency – like tacit consent or a hypothetical contract – the phenomenological strategy draws our attention to the more mundane consideration of what people think. Thus, in a crucial passage at the beginning of the defence of the idea of associative obligations, Horton ‘reminds’ us of what we already know (Horton 2010: 169). To see the force of this strategy of reminding, contrast it with a more elaborated theoretical approach in which abstract concepts, like tacit consent or hypothetical contract, are invoked to explicate the principles of political obligation. Even those leading a reflective social life may have no familiarity with such ideas, which are relatively esoteric matters. By contrast, the sense of belonging and identification that people have with their political community is not esoteric at all. It is a fact of the common moral consciousness of which we may need merely to be reminded. If alternative pictures hold us captive, the reminding may take some effort, but once we have been reminded in this way, we have an immediate grasp of what is meant. A modest method yields substantive results.

From another point of view, however, the phenomenological strategy is a bold one. Here is a problem – the problem of political obligation – that has been with us in one form or another since the days of Socrates. The collection of people who have written about it sounds like a roll call of great and original thinkers of the past: Plato, Sophocles, Aquinas, Hobbes, Locke, Rousseau, Kant, Hegel, Thoreau, T.H. Green up to the leading theorists of our own day. Yet, on the phenomenological strategy, a problem that puzzled and prompted all these minds is going to yield to an analysis in which the scrutiny of ordinary moral consciousness dissolves the problem. I conjecture that it was these bold implications from what appeared to be a modest method that attracted those like Margaret MacDonald (1951) and Thomas McPherson (1967) to the sort of ordinary language analysis – itself a form of phenomenology - by which long-standing philosophical problems were dissolved rather than solved. At the time, the effect of ordinary language analysis was an emancipatory one, since people felt themselves freed from the burdens of traditional theorizing, and, to the extent to which this was an achievement, it was not modest at all. Of course, Horton accepts that conceptual analysis alone will not enable us to derive a theory of political obligation. Nevertheless, he does want to capture the spirit of the conceptual argument (Horton 2010: 143 and 145).

An account of obligation is an account of what *reasons* can be properly offered to people to explain to them why they have any obligations, including political obligations. It follows that, if the associative theory is correct, then it should point to the reasons that can be used to persuade people that they do have political obligations. According to Horton’s account, reflecting on the fact of attachment by those living in a society to that society’s institutions and practices and bearing in mind the virtually unique capacity, by contrast with other associations, of a political community to supply security and order should lead people in general, as well as theorists, to think that they genuinely have reasons for accepting their political obligations. If they understand the two core propositions of associative theory, those inclined to doubt that they have political obligations are by that understanding provided with good reasons to quell their doubts, at least in the central case where individuals are born into a polity. The precise scope of these obligations need not be determined by the philosophical theory of obligations, according to Horton. For example, we cannot infer that all of those over whom a polity asserts its authority are necessarily properly subject to that authority. Nor can we draw much by way of inference to stateless societies or societies that have complex layers of authorities within their system of governance. However, Horton thinks that it is straining too much at the limits of political theory to expect it to settle such matters. Sufficient unto the day is a coherent and plausible account of political obligation in the central, paradigm case.

At this point, however, we can note a tension between Horton’s use of the phenomenological method and his account of the occasion on which the problem of political obligation arises. The phenomenological method relies upon “taken for granted” assumptions. As Horton says, “Where citizens are generally tolerably content with the political arrangements of their society, they may not choose and will not be compelled to think much about their relationship to the political community of which they are members” (Horton 2010: 2). By contrast, the occasions in which the problem of political obligation is theorized are said to be the great crises of identification, from Socrates onwards. In these crises there is deep, if not always widespread, puzzlement and questioning about the grounds of political obligation within societies in which theories of obligation are articulated. Horton notices that there is a tension here, but thinks that he can turn it to his advantage: “Yet, although it is in circumstances such as these that political obligation will most probably be experienced as a *problem*, it is to less troubled times that we should look for an ‘answer’” (Horton 2010: 2), holding that it is ironic that it is in those times when our need for an answer is most urgent that it is most difficult to give an answer.

Yet, is this irony or incompleteness? Behind the claim of irony there is an important ambiguity about the nature of reasons in our understanding of political and social life. To the extent to which human action is action as a result of reasons, to say that someone is acting is to characterise their intention and purposes in acting. However, we can distinguish the role of reasons in helping us understand why someone behaves as they do from reasons as providing agents with considerations having merits as to why they should do what they are going to do. Sometimes this distinction is marked as that between “subjective” and “objective” reasons, but I will mark it simply by the distinction between having reasons and having good reasons, where a good reason is distinguished from a reason by its having some warrant for its adoption. To go beyond phenomenology is to point to this warrant. But why should we need to go beyond phenomenology?

## Beyond Phenomenology?

To explore this question, let us step back from the details of Horton’s own approach to consider the problem of political obligation in general. Political obligation is a special case of moral obligation in general, and the problem of moral obligation was well put by Prichard a century ago:

Any one who, stimulated by education, has come to feel the force of the various obligations in life, at some time or other comes to feel the irksomeness of carrying them out, and to recognize the sacrifice of interest involved; and, if thoughtful, he inevitably puts to himself the question: ‘Is there really a reason why I should act in the ways in which hitherto I have thought I ought to act?’” (Prichard 1912: 1)

On this account the problem of obligation is that the fulfilment of duty is irksome. Duty contrasts with inclination or with a sober calculation of self-interest. Either way, reflection leads actors to question the rational foundations of their obligations. In this situation, the problem, as Prichard formulates it, is whether there a form of reasoning that can be offered to individuals that will make the fulfilment of duty the pursuit of the agent’s own reasoned purposes? In particular, is there any way of showing that the fulfilment of duty is somehow rational in the sense that the agent has good reason to perform that action?

Prichard’s own view was that there was no way to solve the problem apart from recognising the intrinsically binding character of moral obligations. If we do not accept Prichard’s own line of argument, there are two strategies for trying to reconcile prudential self-interest with moral obligation. The first is to deny the premiss that underlies the question; the second is to provide an answer to the question. Accounts of obligation typically appeal to one or other of these strategies. Denying the premiss can take various forms. For some obligation is not irksome, because there is a conceptual connection between being a member of an association and having obligations, so that denying an obligation is like being asked to perform the impossible. You cannot play football without a ball, and, if you try, you have simply failed to understand what it is to participate in the practice of football. For others, denying the premiss involves showing that one’s identity is somehow constituted by one’s membership of a political community, so to deny one’s obligations is to deny some aspect of oneself, an act more irksome that affirming one’s identity through acceptance of the obligation. Yet others deny that there is anything self-evident about acting in one’s own prudent self-interest, a line that was taken by Brian Barry (1989: 285) among others. On this last account, a reason is a reason is a reason. If you have reasons for fulfilling your political obligations, then those reasons are all of a par. There is nothing irksome in acting for the best reasons.

The alternative response to Prichard’s dilemma is to accept that it states a genuine problem, because there is something prima facie odd in agents acting against their own self-interest. Indeed, John Horton (1991: 129) himself noted a similar point in relation to Barry’s dismissal of self-interest, pointing out that, although self-interest cannot provide a complete set of motives, it is always relevant to cite self-interest as a relevant consideration, even if it is outweighed by other considerations. However, it is possible to show why seemingly non-prudential actions can be justified from the point of view of a collectively dependent self-interest. It is this response that takes us beyond phenomenology to the social contract approach.

Suppose a group of people is puzzled as to why they have an obligation to uphold a legal and political order and play their part – however modestly that part may be defined – in the maintenance of their polity. Suppose that their puzzlement is motivated by Prichard’s sense of irksomeness, reflecting their concern for their prudent self-interest. In seeking to address their puzzlement, someone who favours the associative theory of obligation will point out to them that they stand in a special relationship, namely that of membership, to the polity. Members, it will be urged, typically feel a special sense of attachment to their polity. Yet, our putative dissenters may not deny that in some sense they are members; rather they are not moved by that thought and do not share that sense of attachment. They treat their membership as a contingent fact of their biographies, and not by itself a reason to act in accordance with or comply with their obligations. At this point the second strand of the associative argument will come into play and it might be said that the polity in question provides the goods of order and security. Let us suppose that it does. Our dissidents acknowledge this, and accept that these goods are valuable. However, they deny that these facts form a ground of obligation. They find these public goods valuable, but they do not see that they have a prudential reason to contribute to them. They do not see why they should pay their taxes or abide by an inconvenient law if they can avoid doing so without penalty. Moreover, since the distinctive political goods of political association are pure public goods in the standard economists’ sense – that is to say, if they are available to anyone they are available to everyone – there is an opportunity to free ride, and our dissidents do not see why they cannot be free riders. For them the burdens of compliance are greater than the benefits.

The situation of these putative dissenters brings out one feature of the general good of law and order, namely that not only does it have to be supplied by the political association, it also has to be produced by the members of that association, either directly themselves through a willingness to undertake military or citizen service or indirectly through a willingness to contribute to the financing of public goods. The obligation is therefore not merely an obligation to obey the law and customs of the land so far as that is required; it is also an obligation to work so that the organisation and services of the political association can be supplied. On what grounds might individuals be ascribed the relevant obligations to work in this way? What those who are sceptical about fulfilling their obligations because they are irksome need is an argument showing how the logic of cooperative action can be individually beneficial. They need an argument that relates the good of all to the good of each. In saying this, I am not claiming that they will necessarily act on such an argument. That is a matter of whether they can made the rationality of action their motive. But without an argument that stipulates the conditions under which what is collectively rational will also be individually rational, we have no reasons to offer at all.

It is at this point that we need to go beyond the phenomenology of how individuals relate to public goods to a theoretical account of the logic by which those benefits are produced. This is the tradition of Hobbes and the other classical contract theorists, a tradition that has been revived in modern contract theory by the work of Grice (1967) and Gauthier (1986). The key insight within this tradition is that as long as constraints on the freedom to act in one’s self-interest can be rationalised in terms of the interests of the individuals to whom those constraints are applied, then it can validly be said that it is protecting those persons’ own interests. In an early exposition of modern contract theory, Grice (1967: 100) captured this feature well by saying that to claim that some action *X* was prima facie obligatory in a society was to claim that in that society it would be in everyone’s interest to make a contract with everyone else to do *X*. Thus, to take one example, for Grice it would not be true to say of someone for whom it was irksome to keep a particular promise that it was in that person’s interest to do so. However, it would be in their interests to make a contract with everyone else in which promise keeping was prima facie obligatory. In an analogous way, Gauthier frames his account of obligation as one in which society is seen as a co-operative venture to mutual advantage such that “in certain situations involving interactions with others, an individual chooses rationally only in so far as he constrains his pursuit of his own interest or advantage to conform to principles expressing the impartiality characteristic of morality” (Gauthier 1986: 4). Thus, there is a reason – valid to the extent to which it refers to an individual’s share in a common interest that is the ground of his or her own separate interest – for individuals to accept some irksome social obligations. The good of each depends upon the good of all, but only because the good of all is made to depend upon the good of each. This answer takes us beyond the phenomenology of obligation in an intriguing way. It requires us when seeking for reasons for political obligation not only to note the social fact that people generally take it that they have reasons, but also to explore the extent to which these otherwise taken-for-granted reasons are good reasons.

Social contract theory is often thought to presuppose a hypothetical contract, from which actual obligations are inferred from a thought-experiment in which putative choices give rise to obligations. Formulated in this way, there is no reason to suppose that social contract theory can provide an adequate account of obligation. Sam Goldwyn famously said that a verbal contract was not worth the paper that it was written on, and a hypothetical contract seems to take this logic one stage further going beyond spoken words to imagined worlds. Moreover, the development of hypothetical contract theory from the late twentieth century onwards has shown that the conclusions inferred from the contractual thought-experiment are highly sensitive to the assumptions that are made about the circumstances of the imagined contracting parties, so that first-order moral disagreement is simply displaced onto second-order theorizing (Weale 2004).

However, there is an alternative way of conceiving of social contract theory, namely as a way of modelling the logic of social relations that have to hold among individuals in order for a society to be able to function to a tolerable degree. There is an extensive body of social science writing that takes this approach, showing how the legitimate egoistic pursuit of individual advantage needs to be embedded in institutions of collective control if a self-defeating struggle of all against all is to be avoided (Weale 2013: Chapter 2). The argument is that the legitimate pursuit of individual advantage requires the acceptance of certain general norms if that individual advantage is to be realized. Keeping one’s contractual promises may not be individually advantageous on any one occasion, but, unless there were the institution of promise keeping, no one would be able to interact with others to their mutual advantage, and so the pursuit of legitimate individual advantage would fail. To say of a hypothetical thought-experiment that it would be in each person’s interest to make a contract with everyone else to do *X*, under suitable circumstances, is simply to note that persons do best individually in societies in which such social contracts are actually made and enforced and not in societies in which there is an unconstrained pursuit of individual advantage. There are plenty of societies, arguably the vast majority in the modern world, that are trapped in a sub-optimal set of outcomes as a result of individuals seeking to free ride on the contributions of others. A sub-optimal equilibrium is simply one in which individuals do injury to their own interests as a consequence of each trying to outstrip the interests of others. When everyone stands on tiptoe, no one sees any further.

The branch of social science that has done most to explore the logic of collective agreement to common advantage is that concerned with the study of common property resource regimes, unsurprisingly since common property resource regimes are regimes of property management in which securing the fruits of individual labour depends upon the agreed management of resources like fishing stocks, irrigation schemes, forests and grazing rights (see Weale 2013: Chapter 2). In such societies there are typically explicit social contracts that regulate individual access to the common property resource, so that the cumulative consumption of all stays within sustainable limits to the benefit of each. Such contracts are found in societies with very diverse cultures but in circumstances in which each participant to the contract enjoys a rough equality of power with all others, suggesting that what is being expressed in such contracts is not a set of particular cultural values but an underlying logic of individual and collective advantage in circumstances in which all have an incentive to seek their rational good for fear of something worse.

An important element of the social contract in such societies is its highlighting the extent to which individuals typically have two sets of interests. They have first an individual interest in their ability to harvest as much by way of natural resources as possible, and so they are in competition with others. They have second an interest in the integrity and maintenance of the management of the common property, an interest that they have in common with other users (Ostrom 1990: 31). However, these two sets of interests are not discrete. The second condition must be met for the first to be realized. Unless the members of a collectivity can solve their collective action problem, the pursuit of their own particular interests will be frustrated. The fundamental conception of society that lies behind social contract theory is, then, one in which the members of society share certain common interests in a situation of equality of status and power. The corresponding conception of the person that this requires is the ability of persons to have a sense of their own interests, on the one hand, and the ability to make commitments to others to maintain common interests, on the other. From this point of view, a satisfactory account of obligation requires giving individuals some reason to constrain their otherwise free action.

It will be said that this approach does not solve the problem of political obligation, because there will be some people who find that it will pay to be free riders provided that enough of their compatriots are prepared to abide by a collectively prudential set of norms. There is certainly an issue of motivation contained in this problem but it does not affect the rationality of cooperation. If it is genuinely the case that individual advantage in a society is a necessary condition of individuals pursuing their own interests successfully, then it will be true for the putative free riders, and it is therefore in their interests to have made a contract with everyone else in which certain obligations are assumed, even though it may be irksome to fulfil those obligations on certain occasions. Moreover, it is one thing for putative free riders to hope that their shirking of their obligations will not be monitored and observed; it is another thing for them to complain that, when their free riding is observed, their behaviour is punished in order to secure compliance in the future. The desire to avoid playing one’s part in a mutually beneficial scheme of political cooperation cannot be a good reason to avoid the regulation of political authority. Indeed, the empirical evidence from common property resource regimes is that the monitoring of compliance and a graduated system of penalties for non-compliance is an element in their successful functioning (Ostrom, 199: 90). Empirical social contract theory shows us one way in which we might move beyond phenomenology.

## Conclusion: Reflective Equilibirum?

Obviously I have not tried within the limits of this paper to offer a complete alternative account of political obligation to that offered by Horton. There are too many loose ends left for that ambition to be plausible. However, I hope that the sketch of social contract theory explains how we might go beyond the social facts revealed by the phenomenology of identification and membership together with the connection between political association and order and security. Of course, to go beyond phenomenology is not to deny the importance of phenomenology.

In the methodological manifesto that I quoted at the beginning of this paper, Horton contrasts an approach to normative theory in terms of justification from first principles with his own phenomenological approach. This suggests a distinction between the method of reflection and the method of reflective equilibrium. Reflection offers an interpretation of the contents of ordinary moral consciousness, without feeling the need to formulate an abstract theory of social relations in terms of which general principles can be derived through deductive reasoning. However, we should avoid too sharp a distinction between interpretation and abstract deductive argument, particularly if we can identify principles of political order derivable from the general logic of social relations. Some political philosophers who use first principles methods do reject the anchor of the common moral consciousness, denying for example that reflective equilibrium plays a role in their theorizing. Others, like Horton, think that if we stray too far from ordinary moral consciousness we miss the inner point of view from which any satisfactory account of political obligation must be constructed. In this paper I have tried to suggest that an empirically grounded social contract theory interpreted for its normative implications provides a middle way between deductive and interpretative methods. In consequence, we can properly seek for a theoretically justified reflective equilibrium in addition to our interpretative reflections.
